# On the Use of Large Doses of Quinine

**Published:** 1845-07

**Authors:** 


### PRACTICAL MEDICINE, &c.

*On the Use of Large Doses of Quinine.—Report of a Committee of the Medical Department of the National Institute, on Dr. Buck's Paper, "On the Use and Abuse of Medicine."*

(Continued from No. 3.)

The *second* reason why larger doses are more admissible than formerly, is the marked modification of the various diseases to which the human family are liable within the last half century. This is truly remarkable, and it is from this circumstance we must account for the numerous fashions which have prevailed in medicine and medical practice, and which are so pointedly alluded to in the paper of Dr. Buck. Within our own recollection many have reigned, and have been superseded. This, though, may be all accounted for very rationally—the necessity of the change in the mode of practice being required by the changes which disease has undergone; the practice being in accordance with the exigencies of the cases. We must not be understood as expressing the view that the character and the nature of the *semina morborum* have undergone any modification; that there has been really any change in the cause of disease. It is the same causes acting on the *systems* which have been revolutionized by the habits of man, by his advancement in civilization, by his increase of an indulgence in luxuries. These influence the habits, morals and customs of whole nations, and may account for the different influences which the *semina morborum* have now from what they had when man was nearer his primitive state. In illustration of this, we may refer to the influence of climate, season of the year, &c., on the character of disease. We may farther illustrate it by exhibiting to you the fat, jolly, beef-eating and beer-drinking alderman of London; the butler of "my lord's" mansion, confined solely to his domains; or the Frenchman confined to the purlieus of Paris, accustomed to breathe only its foul and polluted air, subsisting on soups, sour wines and "lavements." Compare these with the hardy American, living in an open and well ventilated country, confined to no space, bound by no usage, and subsisting on food compatible with his nature. Now examine what will be the effect of similar causes of disease acting on these two dissimilar classes of individuals. Why, the same cause of disease acting equally on these several individuals would produce dissimilar effects, which would require different methods of treatment.

Here we have illustrated the principle, with which we set out in the above paragraph, that causes modify the action of disease and treatment. Climate, it is well known, produces differences in the character and the type of diseases. It changes the whole nature of the treatment. The subjects of Florida, and of the whole South Western and Southern countries generally, are liable to sudden and violent forms of disease, different in their type from those of the North and East, and generally unknown to those people. This principle, doubtless, accounts for the difference in the quinine practice of the two regions. It accounts for the necessity of giving large doses of the article, in some countries, while smaller doses answer for others. It may account for the fatality attendant on the administration of this medicine in France, and its beneficial effects in this country. This principle, and that already alluded to, viz.: the applicability of this medicine only to periodical malarial diseases, may serve to account for the discrepancy of the testimony relative to the advantages of the large doses of quinine. In speaking of the change which disease has undergone, without assigning other causes for this change than those already mentioned, we may assert our belief that disease has undergone a very essential and marked change in its type in this country. And in this opinion we are not singular. In conversation with men of eminence, we find the same opinion entertained. The nervous system seems to be more or less involved in nearly every form of disease which presents itself to us; and this has been particularly the case in this section of country. Thus we have nervous, neuralgic symptoms complicating nearly every case which presents itself to us. If this fact can be sustained by more extended observation, as we believe it can be, it will go far to account for the modification necessary for the treatment of diseases.

The question now properly presents itself—inasmuch as the mass of beneficial effects of large doses of quinine have been made in the Southern and South Western portions of this country, will the practice equally answer in other sections of the United States? Or should we modify the practice according to the climate, seasons of the year, &c.? Do intermittents of every portion of the United States, and of every country, require to be treated by large doses of quinine? This we consider a question of the first moment. Admitting, as all must do, the propriety of the practice, at least in the South, should it not be imitated elsewhere? What has been the result of the observations of the physicians of the Middle States, and in our own District? Information on this subject, thus far acquired, leads us to the belief, that this class of diseases, arising as it does from the same cause, requires little modification in its modes of treatment. In this city, it is not an uncommon practice to administer 10, 20, or 30 grains of quinine daily, in one, two, or three doses, with decided benefit, not only in intermittent but in neuralgic diseases. This practice, thus pointed out in the paper already alluded to, published in the Baltimore Medical and Surgical Journal, from a highly respectable source in Maryland, is now the common practice of the lower counties of that State. But how shall we meet this question when applied to the Northern sections of the country? Malarial diseases in these are so infrequent, that but few opportunities exist of testing the value of the practice. Judging from the paper of Dr. Buck, we should rather infer, that physicians oppose the practice, either from fear of resorting to it, or ignorant of its advantages. Having succeeded by the continued administration of small doses, they are unwilling to countenance this innovation on established practices. These prejudices are of course to be regarded and duly respected. A sufficient number of observtions have not yet been made, perhaps, to justify the universal adoption of the practice, although sufficient to justify a continuance cf the observations. Time only can prove the value of the practice universally.

And why should not large doses of quinine be preferable to smaller, after all that has been said? Let us now present some reasons drawn from analogy, and from the true *modus operandi* of this medicine. We have stated that all articles of the *materia medica* have their medicinal dose. We may go farther, and assert that the effect of medicines depends often upon the *dose* and the *mode of administration.* Take almost any article of the materia medica and examine its properties; we find that *upon the dose* will depend the effect. Nearly all emetics are tonics in small doses; they act as diaphoretics in other doses, and then we find them producing their specific emetic effects in full doses. Now take *opium;* would you give minute doses to produce sleep in *mania a potu?* Take *calomel;* would you give it in minute doses to produce catharsis? Need we go farther to illustrate, from the materia medica, that upon the dose of a medicine depends its effect? Then why need we exclude from quinine this property of producing different effects, in proportion to its doses? We *should not,* as illustrated by the observations already made. Let us recommend to the profession to cast aside old and wedded prejudices, and to open their minds to conviction. If they are not satisfied with the statements that have been made, and are unwilling to venture upon the administration of large doses of quinine, they may at all events feel assured that no injury can result from a cautious imitation of the practice.—The field is still open for observation and experiment, and the subject is of sufficient importance to demand all the energies of the laborers in science and the friends of humanity. For, after all, it is from the accumulated evidence and experience of the profession, that we are to be governed in this as well as in all points of practice?

It will therefore be seen that we entertain different views as to the administration of large doses of quinine, from our friend Dr. Buck. We agree perfectly in his motto, "*in medio tutissimus,*" &c. We agree with him, also, that medicines are to be used and not abused—"*Utor et non abutor.*" The questions between us, then, are, first, *what is the medium dose,* and what would be the abuse of this medicine? Judging from our own experience, as much good can be derived from 10 to 20 grains as from larger quantities. We would consider 15 grains as a medium dose, though we are not by any means disposed to question the assertions of those who have made more extended observations, as already shown, and who give 30 or 60. We again disagree with the author of this paper, in his opinion that it is improper that such a communication as that of Dr. Van Buren should be placed before the public. On the contrary, we think it should be published. Though an epitome of facts, they were collected after much labor and close observation, by responsible men in the profession, and under the high sanction of the Medical Bureau of the Army. It should be published, because it calls the attention of the profession to a most important subject, one upon which various ideas are entertained by the medical men of this and other countries; thus affording these an opportunity of testing the correctness of the observations.

NOTE.—A good reason for giving large doses of quinine rather than small in intermittent fever, is that a smaller amount of the article is necessary to effect a cure. This I assert on the authority of those who have tested this by many cases. Thus in 75 per cent. a single dose of 20 grains of quinine will effect a cure, while giving it in small doses it will require nearly double the amount. It is a matter also of some importance, inasmuch as this is an unpleasant medicine to take, to diminish the number of doses as much as possible. —*Boston Medical and Surgical Journal.*

